# Supplementary material for: Modeling glioblastoma heterogeneity as a dynamic network of cell states
Source: Mol Syst Biol. 2021 Sep 16;17(9):e10105. doi: 10.15252/msb.202010105 (PMC8444284; doi:10.15252/msb.202010105)
Supplement: Supplementary file 6 — Source Data for Figure 5 [file MSB-17-e10105-s004.zip › Figure5A_sourcedata/GSEA_3017/nosigdb_stateF.GseaPreranked.1622795015644/RICHARDS_NOWAKOWSKI_SCIENCE_2017_IPC_DIV2_UPREG.html]

Details for gene set RICHARDS\_NOWAKOWSKI\_SCIENCE\_2017\_IPC\_DIV2\_UPREG[GSEA]

|  || Dataset | state33017 |
| Phenotype | NoPhenotypeAvailable |
| Upregulated in class | na\_neg |
| GeneSet | RICHARDS\_NOWAKOWSKI\_SCIENCE\_2017\_IPC\_DIV2\_UPREG |
| Enrichment Score (ES) | -0.7222196 |
| Normalized Enrichment Score (NES) | -6.043187 |
| Nominal p-value | 0.0 |
| FDR q-value | 0.0 |
| FWER p-Value | 0.0 |
Table: GSEA Results Summary

  

Fig 1: Enrichment plot: RICHARDS\_NOWAKOWSKI\_SCIENCE\_2017\_IPC\_DIV2\_UPREG      
 Profile of the Running ES Score & Positions of GeneSet Members on the Rank Ordered List

  

| PROBE | GENE SYMBOL | GENE\_TITLE | RANK IN GENE LIST | RANK METRIC SCORE | RUNNING ES | CORE ENRICHMENT || 1 | MYO6 |  |  | 62 | 0.859 | -0.0306 | No |
| 2 | COL4A5 |  |  | 71 | 0.829 | -0.0316 | No |
| 3 | ZNF106 |  |  | 129 | 0.701 | -0.0601 | No |
| 4 | DAAM1 |  |  | 175 | 0.618 | -0.0823 | No |
| 5 | MEGF10 |  |  | 240 | 0.560 | -0.1151 | No |
| 6 | PIK3R3 |  |  | 251 | 0.553 | -0.1184 | No |
| 7 | TMEM47 |  |  | 316 | 0.502 | -0.1515 | No |
| 8 | PSAT1 |  |  | 329 | 0.496 | -0.1561 | No |
| 9 | GPC6 |  |  | 332 | 0.495 | -0.1552 | No |
| 10 | ALDOA |  |  | 377 | 0.479 | -0.1774 | No |
| 11 | PLXNB2 |  |  | 398 | 0.471 | -0.1865 | No |
| 12 | XPC |  |  | 425 | 0.461 | -0.1989 | No |
| 13 | IGF2R |  |  | 470 | 0.444 | -0.2212 | No |
| 14 | CA12 |  |  | 522 | 0.426 | -0.2475 | No |
| 15 | ELOVL2 |  |  | 527 | 0.424 | -0.2480 | No |
| 16 | HDAC9 |  |  | 579 | 0.404 | -0.2743 | No |
| 17 | DENND5B |  |  | 586 | 0.403 | -0.2760 | No |
| 18 | TMSB4X |  |  | 590 | 0.401 | -0.2761 | No |
| 19 | SDC2 |  |  | 596 | 0.399 | -0.2772 | No |
| 20 | TMTC2 |  |  | 619 | 0.392 | -0.2877 | No |
| 21 | GPRC5B |  |  | 653 | 0.385 | -0.3042 | No |
| 22 | SEZ6L |  |  | 657 | 0.383 | -0.3044 | No |
| 23 | NFE2L2 |  |  | 663 | 0.381 | -0.3056 | No |
| 24 | BAZ2B |  |  | 675 | 0.377 | -0.3101 | No |
| 25 | LRRCC1 |  |  | 690 | 0.374 | -0.3163 | No |
| 26 | ZHX3 |  |  | 741 | 0.361 | -0.3422 | No |
| 27 | GABARAP |  |  | 746 | 0.359 | -0.3430 | No |
| 28 | NKAIN3 |  |  | 765 | 0.354 | -0.3514 | No |
| 29 | SIPA1L2 |  |  | 766 | 0.354 | -0.3500 | No |
| 30 | PSD3 |  |  | 788 | 0.349 | -0.3601 | No |
| 31 | SETBP1 |  |  | 790 | 0.349 | -0.3593 | No |
| 32 | C3orf70 |  |  | 794 | 0.348 | -0.3595 | No |
| 33 | TMTC4 |  |  | 795 | 0.348 | -0.3581 | No |
| 34 | LZTS1 |  |  | 811 | 0.345 | -0.3650 | No |
| 35 | DAG1 |  |  | 834 | 0.340 | -0.3757 | No |
| 36 | LAPTM4A |  |  | 854 | 0.335 | -0.3848 | No |
| 37 | ZFHX4 |  |  | 878 | 0.330 | -0.3961 | No |
| 38 | MEGF9 |  |  | 899 | 0.326 | -0.4057 | No |
| 39 | SCRN1 |  |  | 904 | 0.325 | -0.4066 | No |
| 40 | CELSR2 |  |  | 998 | 0.310 | -0.4564 | No |
| 41 | MSI1 |  |  | 1051 | 0.300 | -0.4837 | No |
| 42 | C1GALT1 |  |  | 1076 | 0.296 | -0.4957 | No |
| 43 | SPECC1 |  |  | 1089 | 0.294 | -0.5011 | No |
| 44 | THAP5 |  |  | 1092 | 0.294 | -0.5010 | No |
| 45 | OTX1 |  |  | 1102 | 0.293 | -0.5048 | No |
| 46 | SYPL1 |  |  | 1137 | 0.287 | -0.5223 | No |
| 47 | SEMA5B |  |  | 1147 | 0.285 | -0.5260 | No |
| 48 | SNX3 |  |  | 1150 | 0.285 | -0.5260 | No |
| 49 | LAMA1 |  |  | 1173 | 0.283 | -0.5369 | No |
| 50 | CKB |  |  | 1200 | 0.279 | -0.5501 | No |
| 51 | JAM2 |  |  | 1204 | 0.279 | -0.5506 | No |
| 52 | COX6C |  |  | 1252 | 0.274 | -0.5753 | No |
| 53 | IFNGR1 |  |  | 1289 | 0.269 | -0.5939 | No |
| 54 | NCSTN |  |  | 1315 | 0.267 | -0.6066 | No |
| 55 | ELAVL4 |  |  | 1358 | 0.261 | -0.6286 | No |
| 56 | GAPDH |  |  | 1361 | 0.261 | -0.6286 | No |
| 57 | CSRP2 |  |  | 1386 | 0.258 | -0.6408 | No |
| 58 | WSCD1 |  |  | 1401 | 0.256 | -0.6474 | No |
| 59 | TMEM128 |  |  | 1435 | 0.254 | -0.6645 | No |
| 60 | SUCO |  |  | 1444 | 0.253 | -0.6679 | No |
| 61 | RFC5 |  |  | 1473 | -0.250 | -0.6822 | No |
| 62 | DNA2 |  |  | 1479 | -0.252 | -0.6839 | No |
| 63 | SNRPG |  |  | 1486 | -0.254 | -0.6862 | No |
| 64 | RPA2 |  |  | 1490 | -0.256 | -0.6868 | No |
| 65 | FAM200B |  |  | 1491 | -0.256 | -0.6858 | No |
| 66 | HNRNPA2B1 |  |  | 1503 | -0.261 | -0.6908 | No |
| 67 | BLM |  |  | 1507 | -0.262 | -0.6914 | No |
| 68 | HCFC1 |  |  | 1515 | -0.264 | -0.6942 | No |
| 69 | MSH6 |  |  | 1517 | -0.264 | -0.6937 | No |
| 70 | RBBP8 |  |  | 1518 | -0.265 | -0.6926 | No |
| 71 | ITGB1 |  |  | 1521 | -0.265 | -0.6926 | No |
| 72 | CEP78 |  |  | 1524 | -0.265 | -0.6927 | No |
| 73 | NSMCE4A |  |  | 1533 | -0.267 | -0.6960 | No |
| 74 | ZNF100 |  |  | 1536 | -0.268 | -0.6960 | No |
| 75 | ZNF43 |  |  | 1544 | -0.270 | -0.6987 | No |
| 76 | BAZ1A |  |  | 1547 | -0.271 | -0.6988 | No |
| 77 | RNPS1 |  |  | 1548 | -0.271 | -0.6977 | No |
| 78 | CREB5 |  |  | 1561 | -0.275 | -0.7031 | No |
| 79 | NUP62 |  |  | 1562 | -0.275 | -0.7020 | No |
| 80 | PIN1 |  |  | 1565 | -0.275 | -0.7020 | No |
| 81 | WDHD1 |  |  | 1569 | -0.277 | -0.7026 | No |
| 82 | KHDRBS1 |  |  | 1573 | -0.278 | -0.7031 | No |
| 83 | MRPL51 |  |  | 1576 | -0.279 | -0.7031 | No |
| 84 | PFN1 |  |  | 1582 | -0.282 | -0.7047 | No |
| 85 | IQGAP3 |  |  | 1584 | -0.282 | -0.7041 | No |
| 86 | COX17 |  |  | 1589 | -0.284 | -0.7051 | No |
| 87 | PRKDC |  |  | 1594 | -0.285 | -0.7062 | No |
| 88 | LHX2 |  |  | 1597 | -0.285 | -0.7062 | No |
| 89 | RCC1 |  |  | 1604 | -0.286 | -0.7083 | No |
| 90 | CCDC150 |  |  | 1605 | -0.286 | -0.7071 | No |
| 91 | RPS24 |  |  | 1608 | -0.287 | -0.7071 | No |
| 92 | RPP30 |  |  | 1609 | -0.287 | -0.7059 | No |
| 93 | CNTRL |  |  | 1610 | -0.288 | -0.7048 | No |
| 94 | TMEM97 |  |  | 1629 | -0.293 | -0.7135 | No |
| 95 | HES6 |  |  | 1632 | -0.294 | -0.7134 | No |
| 96 | RSRC1 |  |  | 1633 | -0.294 | -0.7122 | No |
| 97 | GINS1 |  |  | 1634 | -0.295 | -0.7110 | No |
| 98 | SUV39H2 |  |  | 1643 | -0.298 | -0.7142 | No |
| 99 | RPL8 |  |  | 1647 | -0.299 | -0.7147 | No |
| 100 | CEP128 |  |  | 1657 | -0.304 | -0.7184 | No |
| 101 | PARP2 |  |  | 1665 | -0.305 | -0.7210 | Yes |
| 102 | FAM136A |  |  | 1666 | -0.306 | -0.7198 | Yes |
| 103 | ANAPC11 |  |  | 1667 | -0.306 | -0.7185 | Yes |
| 104 | LSM14A |  |  | 1668 | -0.306 | -0.7173 | Yes |
| 105 | ENO1 |  |  | 1673 | -0.308 | -0.7183 | Yes |
| 106 | RDX |  |  | 1678 | -0.310 | -0.7192 | Yes |
| 107 | EIF4EBP1 |  |  | 1681 | -0.312 | -0.7191 | Yes |
| 108 | FADS1 |  |  | 1682 | -0.312 | -0.7178 | Yes |
| 109 | NUP107 |  |  | 1683 | -0.313 | -0.7166 | Yes |
| 110 | TCERG1 |  |  | 1688 | -0.315 | -0.7175 | Yes |
| 111 | DCXR |  |  | 1689 | -0.315 | -0.7162 | Yes |
| 112 | CTPS1 |  |  | 1692 | -0.316 | -0.7160 | Yes |
| 113 | PSMD2 |  |  | 1696 | -0.317 | -0.7164 | Yes |
| 114 | MGME1 |  |  | 1697 | -0.317 | -0.7151 | Yes |
| 115 | ZNF93 |  |  | 1699 | -0.318 | -0.7144 | Yes |
| 116 | RBM17 |  |  | 1702 | -0.319 | -0.7142 | Yes |
| 117 | PRIM1 |  |  | 1703 | -0.320 | -0.7129 | Yes |
| 118 | SNRPC |  |  | 1707 | -0.321 | -0.7133 | Yes |
| 119 | HAUS1 |  |  | 1708 | -0.322 | -0.7120 | Yes |
| 120 | NEDD1 |  |  | 1710 | -0.322 | -0.7113 | Yes |
| 121 | CENPQ |  |  | 1711 | -0.322 | -0.7100 | Yes |
| 122 | PARP1 |  |  | 1713 | -0.323 | -0.7092 | Yes |
| 123 | PHLDA1 |  |  | 1718 | -0.324 | -0.7101 | Yes |
| 124 | CDC25C |  |  | 1724 | -0.327 | -0.7115 | Yes |
| 125 | RALY |  |  | 1729 | -0.330 | -0.7124 | Yes |
| 126 | TCF3 |  |  | 1731 | -0.331 | -0.7116 | Yes |
| 127 | VEZF1 |  |  | 1732 | -0.331 | -0.7103 | Yes |
| 128 | PPP2R3C |  |  | 1736 | -0.335 | -0.7106 | Yes |
| 129 | MCM3 |  |  | 1738 | -0.335 | -0.7098 | Yes |
| 130 | PLK4 |  |  | 1740 | -0.336 | -0.7090 | Yes |
| 131 | RMI1 |  |  | 1743 | -0.337 | -0.7088 | Yes |
| 132 | STIL |  |  | 1744 | -0.337 | -0.7074 | Yes |
| 133 | EXOSC9 |  |  | 1746 | -0.338 | -0.7066 | Yes |
| 134 | XRN2 |  |  | 1750 | -0.340 | -0.7069 | Yes |
| 135 | GLO1 |  |  | 1755 | -0.342 | -0.7077 | Yes |
| 136 | PTBP1 |  |  | 1756 | -0.342 | -0.7063 | Yes |
| 137 | PAICS |  |  | 1757 | -0.342 | -0.7050 | Yes |
| 138 | SNW1 |  |  | 1759 | -0.343 | -0.7041 | Yes |
| 139 | KNOP1 |  |  | 1762 | -0.345 | -0.7038 | Yes |
| 140 | ZNF90 |  |  | 1763 | -0.345 | -0.7024 | Yes |
| 141 | UBTF |  |  | 1764 | -0.346 | -0.7011 | Yes |
| 142 | SUZ12 |  |  | 1766 | -0.347 | -0.7002 | Yes |
| 143 | RPS26 |  |  | 1767 | -0.347 | -0.6988 | Yes |
| 144 | PPM1G |  |  | 1771 | -0.348 | -0.6991 | Yes |
| 145 | RAN |  |  | 1772 | -0.350 | -0.6977 | Yes |
| 146 | HNRNPA3 |  |  | 1776 | -0.351 | -0.6979 | Yes |
| 147 | WDR76 |  |  | 1783 | -0.355 | -0.6998 | Yes |
| 148 | SRPK1 |  |  | 1788 | -0.356 | -0.7005 | Yes |
| 149 | BRIP1 |  |  | 1790 | -0.357 | -0.6996 | Yes |
| 150 | KIF20A |  |  | 1792 | -0.358 | -0.6988 | Yes |
| 151 | ILF2 |  |  | 1795 | -0.360 | -0.6984 | Yes |
| 152 | LIG1 |  |  | 1797 | -0.361 | -0.6975 | Yes |
| 153 | TMEM158 |  |  | 1803 | -0.362 | -0.6988 | Yes |
| 154 | WDR34 |  |  | 1806 | -0.365 | -0.6984 | Yes |
| 155 | GLRX3 |  |  | 1812 | -0.371 | -0.6997 | Yes |
| 156 | KDM1A |  |  | 1814 | -0.371 | -0.6987 | Yes |
| 157 | NEK2 |  |  | 1815 | -0.371 | -0.6972 | Yes |
| 158 | MTMR2 |  |  | 1816 | -0.372 | -0.6958 | Yes |
| 159 | TOMM5 |  |  | 1818 | -0.374 | -0.6948 | Yes |
| 160 | NUP50 |  |  | 1823 | -0.376 | -0.6955 | Yes |
| 161 | RHNO1 |  |  | 1824 | -0.377 | -0.6940 | Yes |
| 162 | XRCC6 |  |  | 1825 | -0.377 | -0.6925 | Yes |
| 163 | GJC1 |  |  | 1829 | -0.379 | -0.6926 | Yes |
| 164 | C18orf54 |  |  | 1830 | -0.380 | -0.6910 | Yes |
| 165 | CDC45 |  |  | 1836 | -0.382 | -0.6923 | Yes |
| 166 | DAZAP1 |  |  | 1837 | -0.383 | -0.6907 | Yes |
| 167 | BARD1 |  |  | 1839 | -0.384 | -0.6897 | Yes |
| 168 | MASTL |  |  | 1843 | -0.385 | -0.6898 | Yes |
| 169 | FANCA |  |  | 1844 | -0.385 | -0.6883 | Yes |
| 170 | FBL |  |  | 1845 | -0.386 | -0.6867 | Yes |
| 171 | MMS22L |  |  | 1846 | -0.387 | -0.6852 | Yes |
| 172 | CCT6A |  |  | 1848 | -0.387 | -0.6842 | Yes |
| 173 | TMA16 |  |  | 1850 | -0.390 | -0.6832 | Yes |
| 174 | SRSF10 |  |  | 1851 | -0.390 | -0.6816 | Yes |
| 175 | CDCA4 |  |  | 1852 | -0.391 | -0.6800 | Yes |
| 176 | PCNT |  |  | 1854 | -0.392 | -0.6790 | Yes |
| 177 | CEP135 |  |  | 1855 | -0.393 | -0.6774 | Yes |
| 178 | ACTL6A |  |  | 1859 | -0.393 | -0.6775 | Yes |
| 179 | KIF18B |  |  | 1860 | -0.393 | -0.6759 | Yes |
| 180 | FANCB |  |  | 1862 | -0.396 | -0.6749 | Yes |
| 181 | E2F8 |  |  | 1864 | -0.396 | -0.6738 | Yes |
| 182 | EXOSC8 |  |  | 1865 | -0.397 | -0.6722 | Yes |
| 183 | POLD2 |  |  | 1868 | -0.398 | -0.6717 | Yes |
| 184 | PPP1CA |  |  | 1872 | -0.399 | -0.6718 | Yes |
| 185 | CHD7 |  |  | 1874 | -0.400 | -0.6707 | Yes |
| 186 | NAE1 |  |  | 1881 | -0.409 | -0.6724 | Yes |
| 187 | CENPO |  |  | 1884 | -0.410 | -0.6718 | Yes |
| 188 | RBMX |  |  | 1885 | -0.412 | -0.6702 | Yes |
| 189 | SHCBP1 |  |  | 1886 | -0.412 | -0.6685 | Yes |
| 190 | RFC4 |  |  | 1887 | -0.413 | -0.6668 | Yes |
| 191 | SEPT10 |  |  | 1888 | -0.414 | -0.6652 | Yes |
| 192 | POLQ |  |  | 1891 | -0.417 | -0.6646 | Yes |
| 193 | YBX1 |  |  | 1892 | -0.417 | -0.6629 | Yes |
| 194 | SAE1 |  |  | 1893 | -0.417 | -0.6613 | Yes |
| 195 | ASF1B |  |  | 1897 | -0.419 | -0.6612 | Yes |
| 196 | RAD18 |  |  | 1898 | -0.419 | -0.6595 | Yes |
| 197 | E2F3 |  |  | 1907 | -0.429 | -0.6622 | Yes |
| 198 | BAZ1B |  |  | 1910 | -0.429 | -0.6616 | Yes |
| 199 | HNRNPH3 |  |  | 1911 | -0.429 | -0.6599 | Yes |
| 200 | ANP32A |  |  | 1912 | -0.430 | -0.6581 | Yes |
| 201 | MNS1 |  |  | 1913 | -0.430 | -0.6564 | Yes |
| 202 | DCP2 |  |  | 1914 | -0.432 | -0.6547 | Yes |
| 203 | POLD3 |  |  | 1918 | -0.436 | -0.6546 | Yes |
| 204 | UHRF1 |  |  | 1921 | -0.438 | -0.6539 | Yes |
| 205 | DIAPH3 |  |  | 1924 | -0.439 | -0.6532 | Yes |
| 206 | HMGXB4 |  |  | 1928 | -0.441 | -0.6531 | Yes |
| 207 | GGCT |  |  | 1930 | -0.442 | -0.6519 | Yes |
| 208 | GINS2 |  |  | 1932 | -0.445 | -0.6506 | Yes |
| 209 | LINC01224 |  |  | 1934 | -0.446 | -0.6494 | Yes |
| 210 | MZT1 |  |  | 1936 | -0.447 | -0.6482 | Yes |
| 211 | SNRPB |  |  | 1937 | -0.447 | -0.6464 | Yes |
| 212 | TTF2 |  |  | 1942 | -0.449 | -0.6467 | Yes |
| 213 | STAG1 |  |  | 1943 | -0.450 | -0.6449 | Yes |
| 214 | PSMA4 |  |  | 1946 | -0.451 | -0.6442 | Yes |
| 215 | CCNF |  |  | 1947 | -0.451 | -0.6424 | Yes |
| 216 | CLIC1 |  |  | 1949 | -0.454 | -0.6411 | Yes |
| 217 | NAP1L1 |  |  | 1951 | -0.455 | -0.6399 | Yes |
| 218 | DDX11 |  |  | 1952 | -0.456 | -0.6380 | Yes |
| 219 | NOP56 |  |  | 1953 | -0.456 | -0.6362 | Yes |
| 220 | RFC2 |  |  | 1954 | -0.457 | -0.6344 | Yes |
| 221 | DDX39A |  |  | 1957 | -0.458 | -0.6336 | Yes |
| 222 | RNASEH2C |  |  | 1964 | -0.462 | -0.6351 | Yes |
| 223 | DNMT1 |  |  | 1969 | -0.465 | -0.6354 | Yes |
| 224 | SNHG1 |  |  | 1973 | -0.469 | -0.6351 | Yes |
| 225 | HILPDA |  |  | 1974 | -0.470 | -0.6333 | Yes |
| 226 | TIMM10 |  |  | 1975 | -0.470 | -0.6314 | Yes |
| 227 | CMC2 |  |  | 1977 | -0.473 | -0.6300 | Yes |
| 228 | CIT |  |  | 1978 | -0.474 | -0.6281 | Yes |
| 229 | C19orf48 |  |  | 1979 | -0.474 | -0.6262 | Yes |
| 230 | CSE1L |  |  | 1985 | -0.479 | -0.6270 | Yes |
| 231 | SUPT16H |  |  | 1986 | -0.479 | -0.6251 | Yes |
| 232 | NASP |  |  | 1988 | -0.481 | -0.6237 | Yes |
| 233 | TIMELESS |  |  | 1989 | -0.482 | -0.6218 | Yes |
| 234 | CDC6 |  |  | 1991 | -0.483 | -0.6204 | Yes |
| 235 | PHIP |  |  | 1992 | -0.483 | -0.6185 | Yes |
| 236 | MCM4 |  |  | 1993 | -0.487 | -0.6165 | Yes |
| 237 | SKA1 |  |  | 1996 | -0.490 | -0.6156 | Yes |
| 238 | SPAG5 |  |  | 1997 | -0.490 | -0.6137 | Yes |
| 239 | PSMC3 |  |  | 1999 | -0.492 | -0.6122 | Yes |
| 240 | FAM111A |  |  | 2002 | -0.497 | -0.6113 | Yes |
| 241 | TK1 |  |  | 2003 | -0.497 | -0.6094 | Yes |
| 242 | LRR1 |  |  | 2004 | -0.497 | -0.6074 | Yes |
| 243 | TUBB6 |  |  | 2006 | -0.500 | -0.6059 | Yes |
| 244 | XPO1 |  |  | 2007 | -0.500 | -0.6039 | Yes |
| 245 | HNRNPUL1 |  |  | 2009 | -0.501 | -0.6024 | Yes |
| 246 | CTCF |  |  | 2010 | -0.502 | -0.6004 | Yes |
| 247 | CDK5RAP2 |  |  | 2012 | -0.503 | -0.5989 | Yes |
| 248 | CHEK1 |  |  | 2013 | -0.504 | -0.5969 | Yes |
| 249 | SIVA1 |  |  | 2014 | -0.505 | -0.5949 | Yes |
| 250 | MAGOHB |  |  | 2015 | -0.505 | -0.5929 | Yes |
| 251 | XRCC5 |  |  | 2016 | -0.507 | -0.5908 | Yes |
| 252 | HIRIP3 |  |  | 2017 | -0.508 | -0.5888 | Yes |
| 253 | FEN1 |  |  | 2018 | -0.511 | -0.5867 | Yes |
| 254 | NT5DC2 |  |  | 2020 | -0.512 | -0.5852 | Yes |
| 255 | SMC1A |  |  | 2021 | -0.513 | -0.5832 | Yes |
| 256 | RRM1 |  |  | 2023 | -0.516 | -0.5816 | Yes |
| 257 | RANBP1 |  |  | 2024 | -0.517 | -0.5796 | Yes |
| 258 | SRRT |  |  | 2025 | -0.517 | -0.5775 | Yes |
| 259 | HAUS8 |  |  | 2026 | -0.517 | -0.5754 | Yes |
| 260 | NME4 |  |  | 2029 | -0.520 | -0.5744 | Yes |
| 261 | PARPBP |  |  | 2030 | -0.520 | -0.5723 | Yes |
| 262 | FABP5 |  |  | 2033 | -0.523 | -0.5713 | Yes |
| 263 | DEPDC1B |  |  | 2037 | -0.527 | -0.5708 | Yes |
| 264 | HELLS |  |  | 2038 | -0.528 | -0.5687 | Yes |
| 265 | CDCA3 |  |  | 2041 | -0.529 | -0.5677 | Yes |
| 266 | CCT5 |  |  | 2042 | -0.529 | -0.5656 | Yes |
| 267 | NCL |  |  | 2045 | -0.532 | -0.5645 | Yes |
| 268 | TTK |  |  | 2047 | -0.533 | -0.5629 | Yes |
| 269 | HNRNPM |  |  | 2048 | -0.533 | -0.5608 | Yes |
| 270 | NCAPD3 |  |  | 2051 | -0.537 | -0.5597 | Yes |
| 271 | ILF3 |  |  | 2052 | -0.537 | -0.5576 | Yes |
| 272 | KIF22 |  |  | 2053 | -0.541 | -0.5554 | Yes |
| 273 | CENPJ |  |  | 2054 | -0.544 | -0.5532 | Yes |
| 274 | SRSF7 |  |  | 2056 | -0.544 | -0.5516 | Yes |
| 275 | SNRPF |  |  | 2057 | -0.545 | -0.5494 | Yes |
| 276 | CTNNAL1 |  |  | 2059 | -0.548 | -0.5478 | Yes |
| 277 | SAMD1 |  |  | 2060 | -0.549 | -0.5456 | Yes |
| 278 | INCENP |  |  | 2061 | -0.556 | -0.5433 | Yes |
| 279 | PTMA |  |  | 2065 | -0.570 | -0.5427 | Yes |
| 280 | DTL |  |  | 2066 | -0.573 | -0.5404 | Yes |
| 281 | FANCD2 |  |  | 2068 | -0.577 | -0.5386 | Yes |
| 282 | ZWINT |  |  | 2069 | -0.577 | -0.5363 | Yes |
| 283 | CEP152 |  |  | 2070 | -0.578 | -0.5340 | Yes |
| 284 | RTKN2 |  |  | 2071 | -0.579 | -0.5316 | Yes |
| 285 | RACGAP1 |  |  | 2072 | -0.579 | -0.5293 | Yes |
| 286 | MND1 |  |  | 2074 | -0.586 | -0.5275 | Yes |
| 287 | LSM4 |  |  | 2076 | -0.587 | -0.5257 | Yes |
| 288 | SKA3 |  |  | 2077 | -0.590 | -0.5233 | Yes |
| 289 | BUB3 |  |  | 2078 | -0.596 | -0.5209 | Yes |
| 290 | AURKB |  |  | 2079 | -0.598 | -0.5185 | Yes |
| 291 | GGH |  |  | 2080 | -0.603 | -0.5161 | Yes |
| 292 | SRSF3 |  |  | 2081 | -0.606 | -0.5137 | Yes |
| 293 | CENPK |  |  | 2082 | -0.607 | -0.5112 | Yes |
| 294 | USP1 |  |  | 2084 | -0.612 | -0.5093 | Yes |
| 295 | PLK1 |  |  | 2087 | -0.624 | -0.5079 | Yes |
| 296 | NCAPD2 |  |  | 2089 | -0.627 | -0.5060 | Yes |
| 297 | SSRP1 |  |  | 2091 | -0.630 | -0.5040 | Yes |
| 298 | MCM8 |  |  | 2092 | -0.635 | -0.5014 | Yes |
| 299 | KIF18A |  |  | 2093 | -0.636 | -0.4989 | Yes |
| 300 | FAM111B |  |  | 2094 | -0.637 | -0.4963 | Yes |
| 301 | SET |  |  | 2095 | -0.639 | -0.4937 | Yes |
| 302 | MIS18A |  |  | 2096 | -0.639 | -0.4912 | Yes |
| 303 | DNAJC9 |  |  | 2097 | -0.641 | -0.4886 | Yes |
| 304 | RFC3 |  |  | 2098 | -0.642 | -0.4860 | Yes |
| 305 | BRCA2 |  |  | 2100 | -0.650 | -0.4840 | Yes |
| 306 | PHF19 |  |  | 2101 | -0.650 | -0.4814 | Yes |
| 307 | ITGB3BP |  |  | 2102 | -0.652 | -0.4787 | Yes |
| 308 | GNG4 |  |  | 2103 | -0.652 | -0.4761 | Yes |
| 309 | NCAPH |  |  | 2104 | -0.652 | -0.4735 | Yes |
| 310 | SNRPD1 |  |  | 2105 | -0.653 | -0.4709 | Yes |
| 311 | PSMC3IP |  |  | 2106 | -0.659 | -0.4682 | Yes |
| 312 | KPNB1 |  |  | 2108 | -0.660 | -0.4661 | Yes |
| 313 | CKAP2L |  |  | 2109 | -0.663 | -0.4635 | Yes |
| 314 | ALYREF |  |  | 2111 | -0.677 | -0.4613 | Yes |
| 315 | RPA3 |  |  | 2112 | -0.678 | -0.4586 | Yes |
| 316 | LSM3 |  |  | 2114 | -0.694 | -0.4563 | Yes |
| 317 | CKS2 |  |  | 2115 | -0.694 | -0.4535 | Yes |
| 318 | RAD21 |  |  | 2118 | -0.697 | -0.4518 | Yes |
| 319 | ANP32B |  |  | 2119 | -0.700 | -0.4490 | Yes |
| 320 | CDCA2 |  |  | 2120 | -0.705 | -0.4462 | Yes |
| 321 | ZNF714 |  |  | 2121 | -0.705 | -0.4434 | Yes |
| 322 | SRSF2 |  |  | 2122 | -0.706 | -0.4405 | Yes |
| 323 | PA2G4 |  |  | 2123 | -0.707 | -0.4377 | Yes |
| 324 | CENPM |  |  | 2125 | -0.709 | -0.4354 | Yes |
| 325 | HNRNPD |  |  | 2126 | -0.713 | -0.4325 | Yes |
| 326 | RAD51AP1 |  |  | 2127 | -0.715 | -0.4297 | Yes |
| 327 | CDCA8 |  |  | 2129 | -0.722 | -0.4273 | Yes |
| 328 | HNRNPAB |  |  | 2130 | -0.729 | -0.4244 | Yes |
| 329 | CKAP2 |  |  | 2131 | -0.729 | -0.4215 | Yes |
| 330 | ATAD5 |  |  | 2133 | -0.742 | -0.4190 | Yes |
| 331 | VRK1 |  |  | 2134 | -0.743 | -0.4160 | Yes |
| 332 | KIF2C |  |  | 2136 | -0.756 | -0.4136 | Yes |
| 333 | BRCA1 |  |  | 2138 | -0.758 | -0.4111 | Yes |
| 334 | CMSS1 |  |  | 2140 | -0.772 | -0.4085 | Yes |
| 335 | KIF14 |  |  | 2141 | -0.773 | -0.4054 | Yes |
| 336 | NDC80 |  |  | 2142 | -0.775 | -0.4023 | Yes |
| 337 | H2AFV |  |  | 2143 | -0.779 | -0.3992 | Yes |
| 338 | FBXO5 |  |  | 2144 | -0.779 | -0.3960 | Yes |
| 339 | KIF23 |  |  | 2145 | -0.782 | -0.3929 | Yes |
| 340 | GMNN |  |  | 2146 | -0.785 | -0.3897 | Yes |
| 341 | LMNB2 |  |  | 2147 | -0.786 | -0.3866 | Yes |
| 342 | MELK |  |  | 2148 | -0.786 | -0.3834 | Yes |
| 343 | ANLN |  |  | 2149 | -0.797 | -0.3802 | Yes |
| 344 | SMC3 |  |  | 2150 | -0.800 | -0.3770 | Yes |
| 345 | KIFC1 |  |  | 2151 | -0.802 | -0.3738 | Yes |
| 346 | DEK |  |  | 2154 | -0.817 | -0.3716 | Yes |
| 347 | DUT |  |  | 2155 | -0.824 | -0.3683 | Yes |
| 348 | NCAPG2 |  |  | 2156 | -0.827 | -0.3650 | Yes |
| 349 | TUBB |  |  | 2157 | -0.831 | -0.3616 | Yes |
| 350 | NUCKS1 |  |  | 2158 | -0.832 | -0.3583 | Yes |
| 351 | LSM5 |  |  | 2159 | -0.835 | -0.3550 | Yes |
| 352 | MCM7 |  |  | 2160 | -0.839 | -0.3516 | Yes |
| 353 | ECT2 |  |  | 2163 | -0.846 | -0.3493 | Yes |
| 354 | HMGB3 |  |  | 2164 | -0.852 | -0.3459 | Yes |
| 355 | KIF20B |  |  | 2165 | -0.852 | -0.3424 | Yes |
| 356 | SPC24 |  |  | 2166 | -0.853 | -0.3390 | Yes |
| 357 | ARHGAP11A |  |  | 2167 | -0.855 | -0.3356 | Yes |
| 358 | DTYMK |  |  | 2168 | -0.858 | -0.3321 | Yes |
| 359 | MCM10 |  |  | 2169 | -0.858 | -0.3287 | Yes |
| 360 | BUB1 |  |  | 2170 | -0.860 | -0.3252 | Yes |
| 361 | FOXM1 |  |  | 2171 | -0.861 | -0.3218 | Yes |
| 362 | CDCA5 |  |  | 2172 | -0.866 | -0.3183 | Yes |
| 363 | LBR |  |  | 2173 | -0.870 | -0.3148 | Yes |
| 364 | BUB1B |  |  | 2174 | -0.871 | -0.3113 | Yes |
| 365 | NUF2 |  |  | 2176 | -0.877 | -0.3083 | Yes |
| 366 | KPNA2 |  |  | 2178 | -0.894 | -0.3053 | Yes |
| 367 | CCDC34 |  |  | 2180 | -0.898 | -0.3022 | Yes |
| 368 | CCNA2 |  |  | 2181 | -0.902 | -0.2986 | Yes |
| 369 | CHAF1A |  |  | 2182 | -0.903 | -0.2950 | Yes |
| 370 | MIS18BP1 |  |  | 2183 | -0.903 | -0.2914 | Yes |
| 371 | SMS |  |  | 2184 | -0.910 | -0.2877 | Yes |
| 372 | KIF15 |  |  | 2185 | -0.915 | -0.2840 | Yes |
| 373 | KIF4A |  |  | 2186 | -0.921 | -0.2803 | Yes |
| 374 | PRR11 |  |  | 2187 | -0.930 | -0.2766 | Yes |
| 375 | ORC6 |  |  | 2188 | -0.937 | -0.2729 | Yes |
| 376 | C21orf58 |  |  | 2189 | -0.937 | -0.2691 | Yes |
| 377 | CENPH |  |  | 2191 | -0.943 | -0.2659 | Yes |
| 378 | CENPW |  |  | 2192 | -0.943 | -0.2621 | Yes |
| 379 | TUBA1B |  |  | 2193 | -0.947 | -0.2583 | Yes |
| 380 | LMNB1 |  |  | 2194 | -0.950 | -0.2544 | Yes |
| 381 | HJURP |  |  | 2195 | -0.962 | -0.2506 | Yes |
| 382 | UBE2T |  |  | 2196 | -0.965 | -0.2467 | Yes |
| 383 | FANCI |  |  | 2198 | -0.966 | -0.2434 | Yes |
| 384 | CDKN3 |  |  | 2199 | -0.979 | -0.2394 | Yes |
| 385 | CCNB2 |  |  | 2200 | -0.983 | -0.2355 | Yes |
| 386 | CKS1B |  |  | 2201 | -0.987 | -0.2315 | Yes |
| 387 | HIST1H1E |  |  | 2202 | -0.992 | -0.2276 | Yes |
| 388 | ESCO2 |  |  | 2204 | -1.004 | -0.2241 | Yes |
| 389 | SMC2 |  |  | 2205 | -1.006 | -0.2200 | Yes |
| 390 | KIF11 |  |  | 2206 | -1.008 | -0.2160 | Yes |
| 391 | EZH2 |  |  | 2207 | -1.017 | -0.2119 | Yes |
| 392 | SPC25 |  |  | 2208 | -1.019 | -0.2078 | Yes |
| 393 | XRCC2 |  |  | 2210 | -1.028 | -0.2042 | Yes |
| 394 | CENPE |  |  | 2211 | -1.036 | -0.2001 | Yes |
| 395 | DHFR |  |  | 2212 | -1.040 | -0.1959 | Yes |
| 396 | NUDT1 |  |  | 2214 | -1.054 | -0.1922 | Yes |
| 397 | MYBL2 |  |  | 2215 | -1.058 | -0.1879 | Yes |
| 398 | CLSPN |  |  | 2216 | -1.060 | -0.1837 | Yes |
| 399 | CENPN |  |  | 2217 | -1.079 | -0.1794 | Yes |
| 400 | UBE2C |  |  | 2218 | -1.081 | -0.1750 | Yes |
| 401 | CENPU |  |  | 2219 | -1.088 | -0.1706 | Yes |
| 402 | H2AFZ |  |  | 2220 | -1.090 | -0.1663 | Yes |
| 403 | HMGB1 |  |  | 2221 | -1.116 | -0.1618 | Yes |
| 404 | NCAPG |  |  | 2222 | -1.122 | -0.1573 | Yes |
| 405 | H2AFX |  |  | 2223 | -1.146 | -0.1527 | Yes |
| 406 | CDK1 |  |  | 2225 | -1.153 | -0.1486 | Yes |
| 407 | PTTG1 |  |  | 2226 | -1.198 | -0.1438 | Yes |
| 408 | ANP32E |  |  | 2227 | -1.200 | -0.1390 | Yes |
| 409 | MAD2L1 |  |  | 2228 | -1.204 | -0.1341 | Yes |
| 410 | GTSE1 |  |  | 2230 | -1.220 | -0.1298 | Yes |
| 411 | DBF4 |  |  | 2232 | -1.247 | -0.1253 | Yes |
| 412 | HMGN2 |  |  | 2233 | -1.267 | -0.1202 | Yes |
| 413 | RRM2 |  |  | 2234 | -1.268 | -0.1151 | Yes |
| 414 | DLGAP5 |  |  | 2235 | -1.273 | -0.1100 | Yes |
| 415 | SMC4 |  |  | 2236 | -1.281 | -0.1049 | Yes |
| 416 | PBK |  |  | 2237 | -1.325 | -0.0996 | Yes |
| 417 | ATAD2 |  |  | 2238 | -1.381 | -0.0940 | Yes |
| 418 | TMPO |  |  | 2239 | -1.382 | -0.0885 | Yes |
| 419 | PRC1 |  |  | 2240 | -1.397 | -0.0829 | Yes |
| 420 | HMGB2 |  |  | 2241 | -1.469 | -0.0770 | Yes |
| 421 | BIRC5 |  |  | 2242 | -1.469 | -0.0711 | Yes |
| 422 | HIST1H1C |  |  | 2243 | -1.485 | -0.0651 | Yes |
| 423 | ASPM |  |  | 2244 | -1.490 | -0.0591 | Yes |
| 424 | TOP2A |  |  | 2246 | -1.562 | -0.0534 | Yes |
| 425 | NUSAP1 |  |  | 2247 | -1.600 | -0.0470 | Yes |
| 426 | TPX2 |  |  | 2248 | -1.609 | -0.0405 | Yes |
| 427 | CENPF |  |  | 2249 | -1.691 | -0.0337 | Yes |
| 428 | HIST1H1D |  |  | 2250 | -1.720 | -0.0268 | Yes |
| 429 | HIST1H1B |  |  | 2251 | -1.796 | -0.0196 | Yes |
| 430 | MKI67 |  |  | 2252 | -1.892 | -0.0120 | Yes |
| 431 | HIST1H4C |  |  | 2253 | -2.984 | 0.0000 | Yes |
Table: GSEA details [plain text format]

  

Fig 2: RICHARDS\_NOWAKOWSKI\_SCIENCE\_2017\_IPC\_DIV2\_UPREG: Random ES distribution      
 Gene set null distribution of ES for **RICHARDS\_NOWAKOWSKI\_SCIENCE\_2017\_IPC\_DIV2\_UPREG**
